# Determinants of Shoot Biomass Production in Mulberry: Combined Selection with Leaf Morphological and Physiological Traits

**DOI:** 10.3390/plants8050118

**Published:** 2019-05-06

**Authors:** Xu Cao, Qiudi Shen, Chunqiong Shang, Honglei Yang, Li Liu, Jialing Cheng

**Affiliations:** 1 College of Biotechnology, Jiangsu University of Science and Technology, Zhenjiang 212003, China; 201600000016@just.edu.cn (X.C.); 162310019@stu.just.edu.cn (Q.S.); 172310025@stu.just.edu.cn (C.S.); 169310001@stu.just.edu.cn (H.Y.); 199700001875@just.edu.cn (L.L.); 2 Key Laboratory of Silkworm and Mulberry Genetic Improvement, Ministry of Agriculture and Rural Areas, Sericultural Research Institute, Chinese Academy of Agricultural Sciences, Zhenjiang 212018, China

**Keywords:** tree biomass, δ^13^C, chlorophyll, leaf phenology, model averaging, *Morus*

## Abstract

Physiological and morphological traits have a considerable impact on the biomass production of fast-growing trees. To compare cultivar difference in shoot biomass and investigate its relationships with leaf functional traits in mulberry, agronomic traits and 20 physiological and morphological attributes of 3-year-old mulberry trees from eight cultivars growing in a common garden were analyzed. The cultivars Xiang7920, Yu711, and Yunsang2 had higher shoot fresh biomass (SFB), which was closely associated with their rapid leaf expansion rate, large leaf area, and high stable carbon isotope composition (δ^13^C). Conversely, the cultivars 7307, Husang32, Wupu, Yunguo1, and Liaolu11 were less productive, and this was primarily the result of slower leaf expansion and smaller leaf size. Growth performance was negatively correlated with leaf δ^13^C and positively correlated with the total nitrogen concentration, indicating that a compromise exists in mulberry between water use efficiency (WUE) (low δ^13^C) and high nitrogen consumption for rapid growth. Several morphological traits, including the maximum leaf area (LA_max_), leaf width and length, petiole width and length, leaf number per shoot, and final shoot height were correlated with SFB. The physiological traits that were also influential factors of shoot biomass were the leaf δ^13^C, the total nitrogen concentration, and the water content. Among the studied leaf traits, LA_max_, leaf δ^13^C, and concentrations of chlorophyll a and b were identified as the most representative predictor variables for SFB, accounting for 73% of the variability in SFB. In conclusion, a combination of LA_max_, leaf δ^13^C, and chlorophyll should be considered in selection programs for high-yield mulberry cultivars.

## 1. Introduction

Fast-growing tree species are arguably the most important feedstock for biomass energy generation. In woody biomass production, most of the attention has been paid to poplar (*Populus*), willow (*Salix*), *Eucalyptus*, and pine (*Pinus*) [1]. However, with the increased demand for energy and the fast-changing global climate, there is an urgent need to identify new candidate fast-growing woody trees that can produce satisfactory biomass under unfavorable climatic and soil conditions [2].

*Morus* species are traditionally best known for their predominant role in rearing silkworm (*Bombyx mori* L.) in the sericulture industry. Mulberry trees have been cultivated perennially in a wide geographical range, from temperate to tropical areas, and their abundance is ascribed to their high adaptability to various agroclimatic conditions [3]. Nowadays, mulberry is receiving more attention because of its immense multipurpose applications, such as fodder additives for livestock [4], as a source of pharmaceutic chemicals [5], and as a pioneer tree species in marginal lands [6]. Remarkably, mulberry plantations can be pruned every 3 months in appropriate agroclimatic situations, and at the same time they thrive well without any loss of growth [7]. The abundance of residual biomass derived from annual pruning operations is an untapped resource that can be used to achieve ecological and energy goals [8]. The above-mentioned beneficial attributes make mulberry a potential energy crop for biofuel generation [9]. Globally, 68 mulberry species have been reported, and over one-third occur naturally in China [3]. A large variation in growth performance and biomass production exists in mulberry, and such variance offers possibilities to select cultivars or genotypes for enhanced biomass production [10].

Physiological traits are the most common indicator to estimate crop biomass production potential. At the leaf level, a large group of growth-related physiological traits, including concentrations of total carbon and total nitrogen [11], water use efficiency (WUE) [12,13], stable isotope compositions of carbon (δ^13^C) and nitrogen (δ^15^N) [14,15], gas exchange rate and chlorophyll fluorescence [16,17], and relative chlorophyll content [18], have been extensively examined in economically important woody crops. These assessments have focused particularly on bioenergy-based genera such as *Populus, Slix*, and *Swida*. Among various physiological traits, leaf δ^13^C is of high significance. It reflects the relationship between plant carbon assimilation and water loss in an integrated manner, and it serves as a robust proxy for instantaneous WUE and may even provide knowledge of the actual WUE if the vapor pressure gradient from the leaf to the air is known [19,20]. Variations in δ^13^C are controlled by genetics and environmental factors, such as temperature, light intensity, ambient CO_2_ concentration, salinity, and soil water and nitrogen availability [21]. Consequently, leaf δ^13^C is a valuable parameter to use when evaluating growth performance and biomass production at the whole-plant level because it serves as an integrated measure of WUE, photosynthetic rate, stomatal conductance, and nitrogen status [22].

Leaf morphological characteristics modulate photosynthetic performance and water balance in plants [23]. Previous studies of fast-growing trees have frequently found a close correlation between tree growth and leaf morphological traits, suggesting that these traits are useful to predict biomass production [24,25]. For instance, in a pot experiment, Marron et al. [14] studied leaf parameters in 31 clones of *P. deltoides × P nigra* growing in a greenhouse, and they proposed that the total leaf area and specific leaf area (SLA) were better indicators of biomass production than leaf growth traits. Nevertheless, Weih et al. [11] compared the shoot biomass production of hybrid willow (*Salix* spp.) in field and pot studies and found that the total leaf area was one of the key traits for the determination of shoot biomass production, irrespective of growth conditions. Furthermore, Müller et al. [17] found that, apart from the total leaf area, the timing of bud burst and the number of total developed leaves were the decisive factors of growth in six aspen (*Populus*) full-sib families. For mulberry, information about the variation in leaf morphological traits among different cultivars in field conditions have not been reported to date. The identification of such traits is important to the development of selection criteria for high-yielding mulberry cultivars or genotypes.

In the present study, we examined shoot biomass-related agronomic traits of eight popular mulberry cultivars. These cultivars were collected from a wide range of original habitats in China, so there are considerable differences in morphological and physiological characteristics. Traits related to water- and nitrogen-use were emphasized given their important roles in plant photosynthesis and growth. These analyses were undertaken on 3-year old mulberry trees grown in a natural environment with uniform water and fertilizer management to maximally highlight the cultivar variability in shoot biomass production. Our goal was to evaluate the variations in morphological and physiological traits among the eight mulberry cultivars and explain how these differences affect mulberry spring shoot biomass production. We hypothesize that the variation in shoot biomass among the investigated mulberry cultivars is closely related to their physiological and morphological traits, which could be used to predict the quantitative yield of spring shoot biomass in mulberry.

## 2. Results

### 2.1. Cultivar Variations in Agronomic Traits

**Figure 1:**
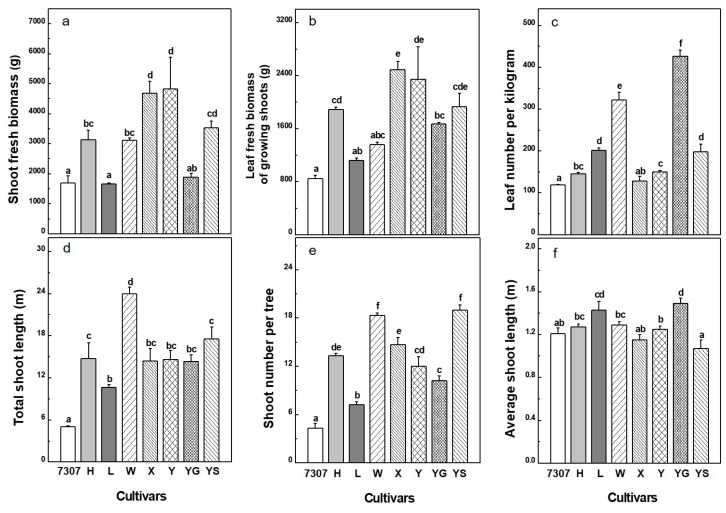
Shoot biomass-related agronomic attributes including shoot fresh biomass (**a**), leaf fresh biomass of growing shoots (**b**), leaf number per shoot (**c**), total shoot length (**d**), shoot number per tree (**e**), and average shoot length (**f**) of the eight 3-year-old mulberry cultivars: 7307, Husang32 (H), Liaolu11 (L), Wupu (W), Xiang7920 (X), Yu711 (Y), Yunguo1 (YG), and Yunsang2 (YS). For each cultivar, the presented data are the means ± SE (n = 3–4). Different letters on the error bar indicate significant differences between cultivars.

Significant differences were detected among the tested mulberry cultivars in shoot fresh biomass (SFB), leaf fresh biomass of growing shoots, leaf number per kilogram, total shoot length, shoot number per tree, and average shoot length (Figure 1). The means of SFB differed up to 3-fold among the eight cultivars, ranging from 1666.92 ± 27.93 to 4681.03 ± 398.33 g. Liaolu11 and 7307 had the lowest biomass production, while Xiang7920 and Yu711 had the highest (Figure 1a). The pattern of variation in leaf fresh biomass of growing shoots was similar to the variation pattern in SFB and ranged from 843.85 ± 55.05 to 2484.65 ± 124.45 g (Figure 1b). Leaf number per kilogram varied from 119 ± 11 to 427 ± 14, indicating that there could be a marked difference in leaf morphological characteristics among the tested cultivars (Figure 1c). Total shoot length ranged from 4.99 ± 0.18 to 23.95 ± 0.98 m. The cultivar 7307 had the shortest shoot length and Wupu had the highest (Figure 1d). Shoot number per tree, ranging from 4 to 19, showed a similar variation pattern to that of total shoot length, and the two cultivars Wupu and Yunsang2 had the most shoots (Figure 1e). The average shoot length ranged from 1.07 ± 0.08 to 1.49 ± 0.05 m in the eight cultivars, among which Xiang7920 and Yunsang2 had the shortest shoot, while Yunguo1 and Liaolu11 had the longest shoot (Figure 1f). The diverse above-ground biomass production capacity among the studied mulberry cultivars suggests that considerable variations in shoot morphology and leaf physiology could be involved.

### 2.2. Variations in Shoot Morphological Traits

**Figure 2:**
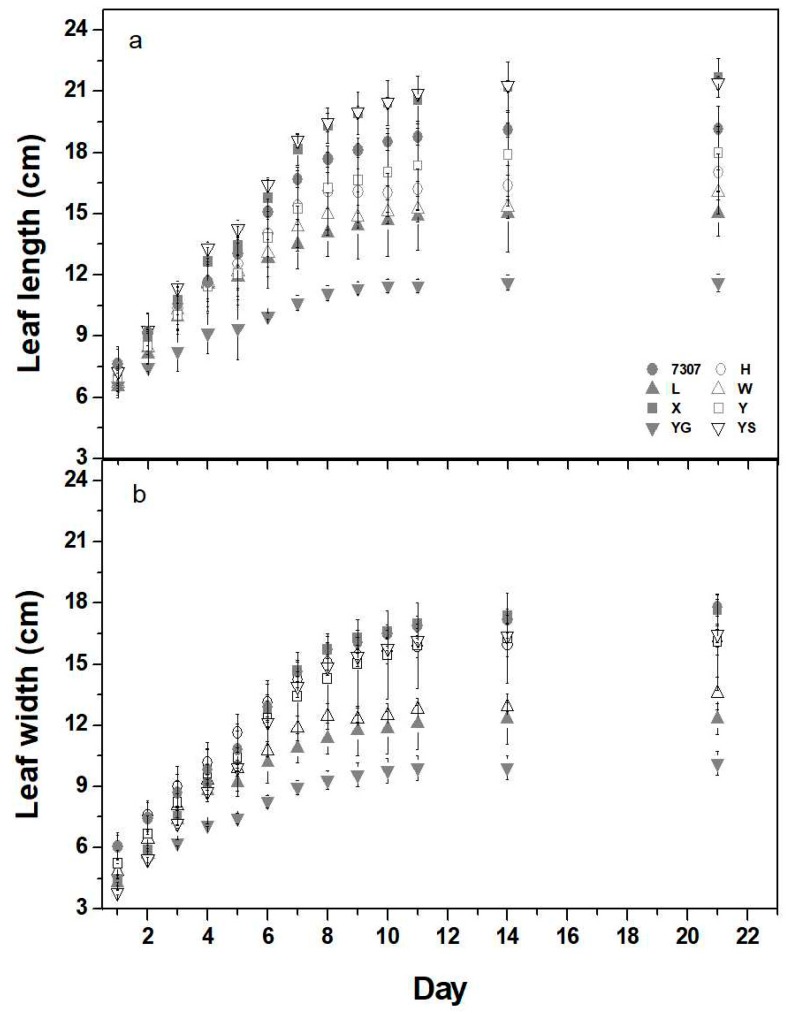
Expansion of leaf length (**a**) and leaf width (**b**) in eight 3-year-old mulberry cultivars: 7307, Husang32 (H), Liaolu11 (L), Wupu (W), Xiang7920 (X), Yu711 (Y), Yunguo1 (YG), and Yunsang2 (YS). Measures were performed on the third leaf and were initiated when the leaf finished unfolding and was beginning to expand. For each cultivar, the presented data represent the means ± SE of measurement of 3–4 trees.

Leaf development was initially monitored on the third lamina in the apex when the leaf had just finished unfolding and was beginning to expand. In the tested mulberry cultivars, active daily leaf expansion was observed in both length and width in the following 8 days. The expansion progressed further for 5 days at a very gentle pace until the leaves reached their full, mature size after a total duration of 21 days (Figure 2). The rate of leaf expansion was calculated from measurements taken in the first 9 days given the strong linear increase in both length and width (R^2^ > 0.95) during this period. The cultivars Xiang7920 and Yunsang2 showed the highest expansion rate in length (ca. 1.7 cm day^−1^) and width (ca. 1.5 cm day^−1^), while the cultivar Yunguo1 achieved a mean of only 0.6 cm day^−1^ in length and 0.7 cm day^−1^ in width, and the other cultivars varied between these extremes (Table 1). Mature leaf size (maximum leaf length and width) was the largest in the cultivar Yunsang2 (24.9 and 21.0 cm) and lowest in the cultivar Yunguo1 (15.1 and 11.3 cm). The cultivars Xiang7920 and Yu711 also showed a comparable leaf size to that of Yunsang2 (Table 1). Maximum leaf area (LA_max_) differed by more than 3-fold, ranging from 104.6 to 343.6 cm^2^, with the highest LA_max_ observed in the cultivars Yu711 and Yunsang2 and the lowest in the cultivar Yunguo1. Specific leaf area of the largest leaf (SLA_max_) varied from 161.9 to 235.5 cm^2^ g^−1^, with Yu711 having the lowest SLA_max_ and Liaolu11 having the highest. Additionally, the cultivars Xiang 7920, Yu711, and Yunsang2 had the thickest petioles (4.53–4.83 mm), which were approximately two times thicker than that of Yunguo1 (2.63 mm). Maximum petiole length ranged from 3.7 to 7.6 cm and displayed a variation pattern similar to the variation in maximum petiole width (Table 1).

Spring growth before summer pruning was significantly different among the tested cultivars. The shoot height and diameter that developed during spring were the highest in the cultivar Liaolu11, whereas the lowest values were in Yunsang2, presenting an approximately 60% difference (Table 1). This was in good accordance with the variation pattern in their average shoot length (Figure 1f). Leaf number per shoot among the mulberry cultivars was the highest in Liaolu11 and Yunsang2, and lowest in Husang32.

### 2.3. Variations in Leaf Physiological Traits

**Figure 3:**
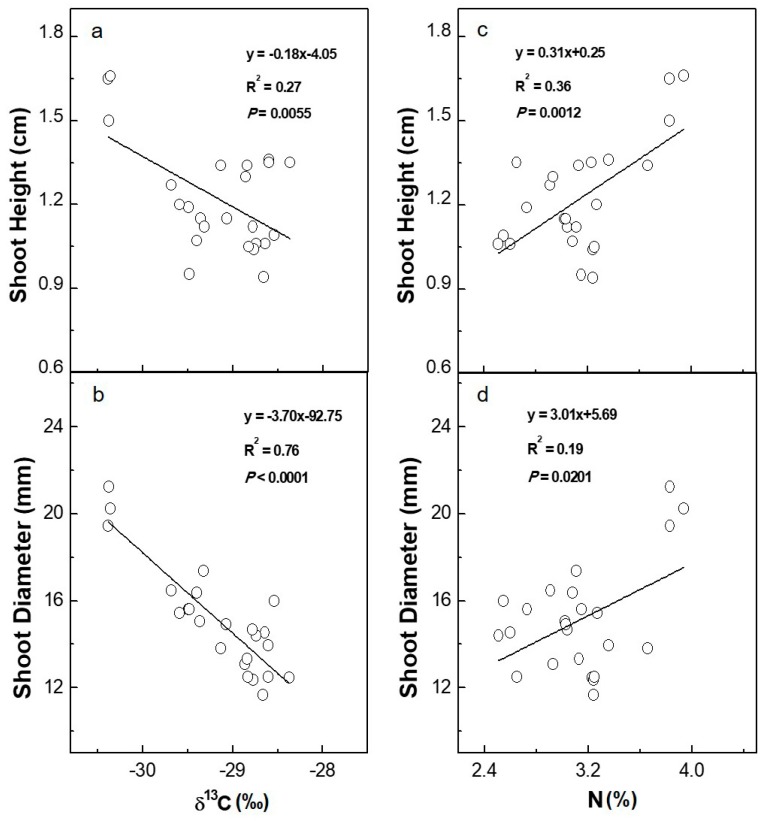
Stable carbon isotope (δ^13^C, ‰) and total nitrogen concentration (N, %) and their relationships with the height (**a**,**c**) and diameter (**b**,**d**) of shoots in eight 3-year-old mulberry cultivars, three individual trees were measured for each cultivar. Each symbol is the value for one plant per cultivar. The linear regression equations for correlating shoot height and shoot diameter with δ^13^C are y = 0.18x − 4.05 (R^2^ = 0.27, *p* = 0.0055) and y = -3.70x + 92.75 (R^2^ = 0.76, *p* < 0.0001), respectively. The regression equations for correlating shoot height and shoot diameter with N are y = 0.31x + 0.25 (R^2^ = 0.36, *p* = 0.0012) and y = 3.01x + 5.69 (R^2^ = 0.19, *p* = 0.0201), respectively.

**Figure 4:**
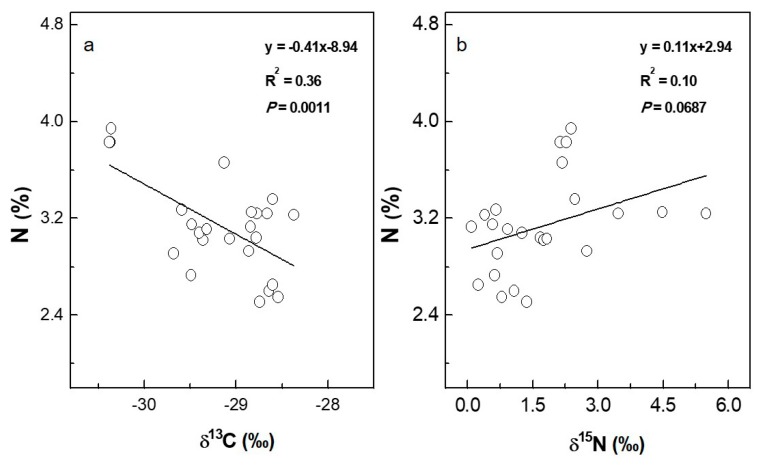
Relationships between leaf total nitrogen concentration (N, %) and stable isotopes of either carbon (δ^13^C, ‰) (**a**) or nitrogen (δ^15^N, ‰) (**b**) in eight 3-year-old mulberry cultivars, three individual trees were measured for each cultivar. Each symbol is the value for one plant per cultivar. The linear regression equations for correlating δ^13^C and δ^15^N with N are y = 0.41x − 8.94 (R^2^ = 0.36, *p* = 0.0011) and y = 0.11x + 2.94 (R^2^ = 0.10, *p* = 0.0687), respectively.

The range of stable carbon isotope composition (δ^13^C) in the mature leaves of the eight 3-year-old mulberry cultivars was from −30.37 to −28.60‰ (Table 2). Less positive values of δ^13^C were detected in cultivars (e.g., Liaolu11) with both higher shoot height and shoot diameter and vice versa (Figure 3). The linear regression model between shoot height and δ^13^C demonstrated that 27% of the variability (R^2^) in shoot height could be explained by leaf δ^13^C (*p* < 0.01, Figure 3a). Furthermore, a pronounced negative association was observed between shoot diameter and δ^13^C. Up to 76% of the variability in shoot diameter was attributed to leaf δ^13^C (*p* < 0.0001, Figure 3b). The total carbon concentration in leaves showed a small variation, with means ranging from 39.73 to 44.00%, and only the cultivars 7307 and Xiang7920 exhibited significant differences compared with the other cultivars (Table 2). Values of stable nitrogen isotope composition (δ^15^N) in mature leaves ranged from 0.21 to a maximum of 4.40‰ among the tested cultivars, with Yunguo1 having the lowest value and Yunsang2 having the highest (Table 2). As an important nutritional element, total nitrogen concentration ranged from 2.55 to 3.87%, with the lowest value found in Xiang7920 and the highest detected in Liaolu11 (Table 2). A significant relationship (*p* < 0.01, R^2^ = 0.36) was found between shoot height and total nitrogen concentration (Figure 3c). Larger values of shoot height were associated with higher total nitrogen concentration in mulberry leaves. Likewise, a less close yet significant correlation (*p* < 0.05, R^2^ = 0.19) was detected between shoot diameter and total nitrogen concentration (Figure 3d). Furthermore, the regression model also presented a significant relationship between total nitrogen concentration and δ^13^C (*p* < 0.01, R^2^ = 0.36). Higher total nitrogen concentration values were recorded in cultivars with more depleted values of δ^13^C (Figure 4a). Otherwise, there was no significant correlation between total nitrogen concentration and δ^15^N (*p* >0.05, R^2^ = 0.10) (Figure 4b).

Leaf water content ranged from 67.9 to 73.7% among the tested mulberry cultivars (Table 2). It was the highest in 7307 and the lowest in Yunsang2. The content of photosynthetic pigments in mature leaves of the eight mulberry cultivars displayed significant differences (Table 2). Chlorophyll a content (chla) was the highest in Wupu (1.70 mg g^−1^ Fw) and the lowest in Husang32 (1.19 mg g^−1^ Fw). A similar variation pattern was found for chlorophyll b content (chlb), and the two chlorophylls together resulted in the highest chlorophyll content in Wupu and the lowest in Husang32 (Table 2). The content of carotenoid in leaves showed a large variation, with means ranging from 4.23 to 108.40 μg g^−1^ Fw. Yunsang2 showed the highest carotenoid content while Husang32 had the lowest (Table 2).

### 2.4. Shoot Biomass Analysis

**Figure 5:**
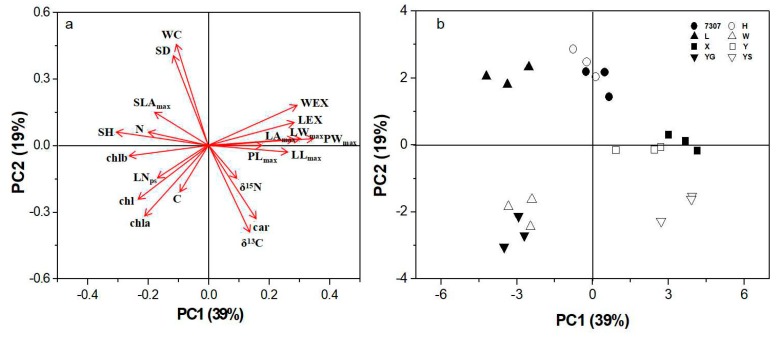
Principal component analysis (PCA) plots of morphological and physiological traits (**a**) and the distribution of the eight studied mulberry cultivars (**b**) in the orthogonal plane (PC1 × PC2).

Of all the functional parameters assayed, the leaf fresh biomass of growing shoots correlated best with SFB (*p* < 0.001, r = 0.89), while the relationships with other morphological (LA_max_, LL_max_, LW_max_, PL_max_, PW_max_, LN_ps_, and SH) and physiological traits (WC, δ^13^C, and N) were less close but statistically significant (Table 3). All the functional parameters were analyzed using principal component analysis (PCA), which reduced the dimensionality of the data set by extracting the predominant components associated with cultivar variations. As shown in Figure 5a, the first two planes of the PCA together accounted for approximately 58% of the variability in all the investigated traits among the eight cultivars (PC1: 39%, PC2: 19%). Axis PC1 was mainly driven by multiple correlated morphological traits (PW_max_, LW_max_, WEX, LL_max_, LEX, LA_max_ and SH) and two physiological traits (chl, chlb), as indicated by their high loading values (Table S1). On the contrary, axis PC2 was captured primarily by physiological traits, including foliar water content (WC), carotenoid content (car), δ^13^C and chla and one morphological trait, which was shoot height (SH). The location of the cultivars in the PCA ordination space showed a clustered pattern in some cultivars (Figure 5b). The cultivars Xiang7920 and Yu711 were clustered in leaf development; otherwise, the cultivars Wupu and Yunguo1 appeared to be distinct in new leaf appearance, photosynthesis, and carbon accumulation compared with the other cultivars. The cultivar Yunsang2 invested more in water use efficiency and nitrogen assimilation, while Liaolu11 maintained a relatively high shoot diameter and foliar water content. The cultivars 7307 and Husang32 tended to show intermediate levels in morphological and physiological traits.

**Figure 6:**
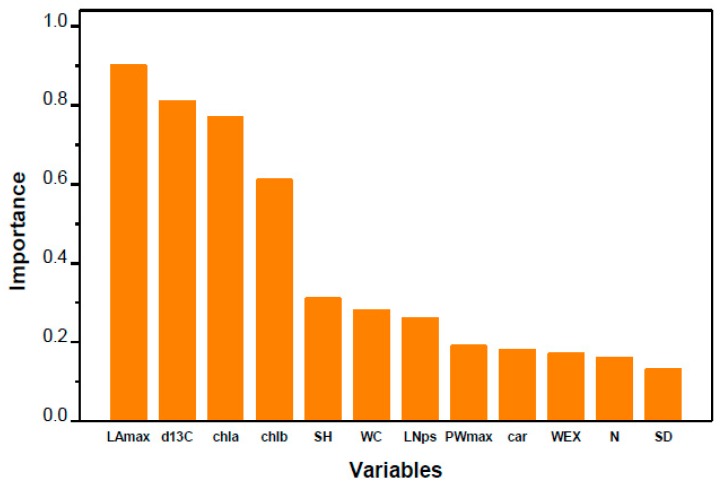
Relative importance values (Akaike weights) of the predictors using model averaging (MuMIn package in R). LA_max_: maximum leaf area; PW_max_: maximum petiole width; WEX: leaf width expansion rate; LN_ps_: leaf number per shoot; SD: shoot diameter; SH: shoot height; WC: foliar water content; δ^13^C: stable carbon isotope composition; N: total nitrogen concentration; chla: chlorophyll a content; chlb: chlorophyll b content; car: carotenoid content.

Key functional traits that could represent the variability in spring yield (expressed as SFB) were identified by only selecting the physiological and morphological traits with no or weak relationships as candidate variables for model averaging. In total, 12 traits were used, and they were generally independent of each other within the same functional group (Figure S1), which was suitable for further analysis by model averaging. Model averaging revealed that the most important variables for shoot biomass were LA_max_, δ^13^C, chla, and chlb, with their calculated importance values ranging from 0.61 to 0.90 (Figure 6). The multiple regression model constructed with these 4 variables could explain 73% (Table 4, Multiple R^2^ = 0.7287) of the variability in shoot biomass in mulberry. Notably, LA_max_ and δ^13^C were recognized as the most significant contributors to this model (Table 4, *p* < 0.001).

## 3. Discussion

### 3.1. Cultivar Variation in Shoot Biomass and Related Functional Traits

The spring shoot biomass of mulberry plantations is important for the sericulture industry because there is a huge demand for mulberry leaves for raising silkworm in spring [26]. The eight cultivars could be roughly divided into two groups according to their shoot biomass. The high shoot biomass group included the cultivars Xiang7920, Yu711, and Yunsang2, while the rest of the cultivars, Husang32, 7307, Liaolu11, Yunguo1, and Wupu, were included in the low shoot biomass group (Figure 1a). These two groups had dramatically different morphological and physiological features that led to differences in shoot biomass. The three high-yielding cultivars were not competitive in shoot growth (i.e., height, diameter, and leaf number per shoot), but they had relatively higher values in foliage production, as indicated by the leaf expansion rate in length and width (Table 1). Because the number of days to achieve mature leaf size was generally similar in the examined cultivars (Figure 2), the rapid leaf expansion rate translated into significantly higher values for the maximum size of leaf lamina and petiole and the largest individual leaf area (LA_max_) (Table 1). In contrast, the cultivars Wupu and Yungguo1, although having faster new leaf appearance and relatively higher chlorophyll content, displayed lower shoot biomass because of their slower leaf expansion rate and smaller leaf size (Table 1 and Table 2). The cultivar Liaolu11 exceeded the other cultivars in shoot growth performance, and this corresponded well with it attaining the highest leaf total nitrogen concentration. However, this cultivar was the weakest in shoot biomass. The discrepancy between shoot biomass and shoot growth in Liaolu11 is probably due to it having the lowest shoot number per tree and the highest SLA_max_ (Figure 1e, Table 3), which often indicates thinner leaves [27]. The cultivars 7307 and Huang32 had moderate performance in both physiological and morphological traits. Therefore, it is anticipated that the great variability in shoot biomass among the tested cultivars is the outcome of the diversity in both physiological and morphological traits, although the latter appears to be more influential. This is supported by the PCA results, which indicated that morphological traits accounted for a higher proportion of the variation in shoot biomass production (Figure 5, 39% vs. 19%). Larger genetic variation in morphological traits than physiological traits has been documented in other similar comparative investigations, which generally echo our results [17,28].

### 3.2. Leaf Physiological Traits Predicting Shoot Biomass

Among the six yield-related agronomic traits, shoot fresh biomass has the highest economic value in sericulture practice. Previous studies on various plant species have demonstrated that several physiological attributes centered around carbon and nitrogen metabolism, photosynthesis, and chlorophyll fluorescence are important factors influencing biomass production [11,14,16,18]. Because of the importance of carbon resources for plant biomass production, δ^13^C was determined to estimate water use efficiency (WUE) in the examined cultivars. WUE, the ratio of carbon assimilated to water consumed, has been extensively found to be significantly correlated with δ^13^C in various plant species [20,27,29], but caution is warranted in the interpretation of δ^13^C as a measure of WUE [30]. Nevertheless, it is assumed that leaf δ^13^C could be a useful indication of broad categories of soil water availability and plant physiological water use efficiency in different tree species [31]. Higher δ^13^C is particularly important for plant survival under drought conditions to reduce transpiration [32], as indicated by the higher δ^13^C values in seedlings from water-limited zones than those from wet sites [33,34]. Variations in δ^13^C in natural growing conditions have been reported for various tree species [27,35,36] other than mulberry. The range of δ^13^C values detected in the tested mulberry cultivars was comparable to that reported for broadleaved tree species growing in natural environments (Table 2). Notably, the cultivars Wupu, Xiang7920, Yunguo1, and Yunsang2 had relatively higher δ^13^C, and this corresponds well to their original habitats, which experience water shortages with high frequency and severity. It is cautiously inferred that these cultivars might gain more advantages from greater water use relative to biomass accumulation than the other cultivars under drought conditions. Many studies have demonstrated that changes of δ^13^C could be closely tied to drought tolerance in trees [37,38]. Further investigations into the growth and/or biomass production capacity of these high-δ^13^C cultivars under water limited conditions are needed.

In this study, δ^13^C was positively correlated with shoot biomass (Table 3) and negatively correlated with growth performance (final shoot height and diameter at the end of the spring growing season) among the tested mulberry cultivars (Figure 3a,b). The power of δ^13^C was further confirmed by its characterization as the most important and significant physiological predictor variable for shoot fresh biomass production using model averaging (Figure 6, Table 4), the results of which highlighted the close relationship between water use efficiency and shoot biomass in mulberry. While extensive studies have addressed the relationship between δ^13^C and growth in herbaceous crops, such investigations in woody plants are limited and are still inconclusive because of the frequently reported contradictory results. Verlinden et al. [39] found that growth traits were poorly linked to δ^13^C in a poplar plantation consisting of six poplar genotypes, and they proposed the potential for screening genotypes with both high WUE and high growth. Ingwers et al. [40] detected a significant negative correlation between δ^13^C and above-ground current annual biomass in old loblolly pine (*Pinus teada* L.). However, a positive relationship between growth (height and stem diameter) and foliar δ^13^C is more frequently reported [12,13,41], suggesting that faster growth might be associated with higher WUE. In particular, Jannatul Fardusi et al. [22] performed a meta-analysis to investigate the average patterns of the relationships between multiple growth attributes and δ^13^C. They found that biomass and height were globally and positively correlated with δ^13^C on the basis of data from 34 species from 16 genera. However, it was emphasized that the positive relationship was established mainly for coniferous trees at the juvenile stage under suboptimal or controlled conditions [22]. Therefore, the influence of tree age and environmental conditions on biomass production should not be underestimated. In the current study, the positive relationship between δ^13^C and biomass production is in accordance with previous results. On the other hand, the negative relationship between δ^13^C and growth suggests that stomatal conductance takes control of WUE, which allows for a higher influx of CO_2_ into the mesophyll cells. The increased intercellular CO_2_ concentration enhances the photosynthetic rate, thereby leading to high growth concurrently. Consequently, a lower WUE but a better growth performance is expected. Similar to our results, a negative correlation between tree size and δ^13^C was identified in *Aquilaria crassna*, a tropical tree species [15], which suggests the strategic sacrifice of WUE for high growth performance. In areas where water is not a limiting factor on most occasions, such as Eastern China, high stomatal conductance contributes to higher photosynthetic rates and growth, so trees do not have to be economic in WUE since there is a low risk of water deficit.

In addition to carbon and water, nitrogen is an important nutritional element for photosynthetic capacity and growth in trees [42,43]. δ^15^N is determined by both the isotope ratios of external nitrogen forms (NO_3_^−^, NH_4_^+^ or amino acids) and physiological mechanisms within plants [44]. For co-occurring plants, nitrogen isotope fractionation during nitrogen absorption, assimilation, and cycling can yield information about differences in patterns of nitrogen acquisition efficiency [45]. The significantly different foliar δ^15^N in the tested mulberry cultivars indicates considerable variation in nitrogen use capacity, although no correlation was detected in this study between foliar δ^15^N and shoot biomass. The leaf total nitrogen concentration among the inspected mulberry cultivars varied from 2.55 to 3.87%, with an average value that was slightly lower than that of mulberry trees with proper nitrogen management [46], but not under threat of nitrogen deficiency. Correspondingly, a positive correlation between leaf nitrogen concentration and shoot growth performance was detected among the examined mulberry cultivars in this study (Figure 3c,d). The positive relationship indicates that the growth of mulberry trees could increase as the leaf total nitrogen concentration increases when the soil nitrogen supply is not in excess. In contrast, the absence of a positive correlation has been reported for mulberry when the native fertility of the soil at the growing site was sufficient [47] or nitrogen fertilizer was overdosed [48]. The tree growth rate could even be repressed because excess nitrogen has been found to impair photosynthesis and cause an imbalance in nutritional elements [49,50], which could also suppress a plant’s defense capacity to potential threats from insect pests and/or disease [51,52]. Nevertheless, this positive relationship did not translate to high shoot biomass production in this study (Table 3), and the contribution of leaf total nitrogen concentration to the variation in final biomass production was quite small (Figure 6). This is in agreement with the results of a previous study on hybrid willows that negated the feasibility of adopting leaf total nitrogen concentration as a determinant of biomass production [11]. Leaf total nitrogen concentration is, on the one hand, the realized balance of biomass accumulation and nutrient reserve, and both processes might be partly independent in time and regulated by different environmental factors [53]. On the other hand, the remobilization and allocation of nitrogen can vary greatly under natural conditions [54]. As a consequence, leaf total nitrogen concentration is assumed to be a poor predictor for long-term biomass production.

In this study, leaf chlorophyll a and chlorophyll b, although not found to directly relate to SFB (Table 3), were identified as physiological predictor variables for shoot biomass (Figure 6, Table 4). Chlorophyll, residing in the thylakoid membranes of the chloroplasts, is responsible for light absorption and transformation to drive photosynthesis in green tissues, and hence, strongly affects tree biomass production [55]. However, the adoption of chlorophyll as a predictor variable for shoot biomass production has not been proposed. Among the tested cultivars, the cultivars Liaolu11, Wupu, and Yunguo1 had higher chlorophyll content, which corresponded well to their higher leaf total nitrogen concentration compared with the other cultivars (Table 2). In forestry, chlorophyll content has been frequently used as a proxy for leaf photosynthetic nitrogen concentration [56] and leaf maximum carboxylation rate [57] to evaluate carbon and water cycles. Furthermore, a positive correlation between community-level chlorophyll content and gross primary productivity has been detected in a wide range of environmental conditions and various species at the ecological scale [58]. The present finding emphasizes the significant contribution of chlorophyll content to the estimation of shoot biomass production in mulberry trees, which might especially be useful for tree species with large leaves.

### 3.3. Leaf Morphological Traits Predicting Shoot Biomass

Both the correlation analysis and model averaging revealed the significance of individual largest leaf area (LA_max_) as the best predictor variable for shoot biomass production in mulberry, surpassing all of the other evaluated parameters (Figure 6, Table 4). Leaf morphological characteristics have been recognized to modulate photosynthetic performance and water balance in plants [23]. Previous studies on fast growing-trees have frequently found a close relationship between growth and some leaf morphological traits, such as total leaf area, specific leaf area, leaf area ratio, petiole length, leaf number, leaf duration, and new leaf emergence, depending on the species, growth conditions, and tree age [24,25]. It has been illustrated that productivity differences between closely related aspen (*Populus tremuloides*) species are largely ascribed to differences in morphological traits [17], which matches our results. Although candidate morphological determinants of biomass production may vary in different circumstances, total leaf area appears to be valid as a key trait for the determination of shoot biomass production in either controlled conditions or natural fields [11]. Nevertheless, some other reports have documented the feasibility of using LA_max_ rather than total leaf area to assess biomass production, although the relative predictive power might be different for various environmental conditions, especially for fast-growing tree species, such as poplar [16] and agarwood [15]. Compared with total leaf area, LA_max_ is easier to assess, and it can be regarded as an integrated measure of leaf growth and development (e.g., leaf expansion, new leaf emergence, light interception and hence photosynthesis) and even whole-plant performance [59]. For mulberry trees, which develop large leaves similar to non-woody species, maximum leaf area could be a better indicator of tree biomass.

Taken together, the agronomic, morphological, and leaf physiological traits of the eight examined mulberry cultivars showed large genetic variability. The cultivars Xiang7920, Yu711, and Yunsang2 had higher shoot fresh biomass, which was closely associated with their rapid leaf expansion rate, large leaf area, and high δ^13^C. Conversely, the cultivars 7307, Husang32, Wupu, Yunguo1, and Liaolu11 were relatively less productive, primarily because of their slower leaf expansion and smaller leaf size. Growth performance (shoot height and diameter) were negatively correlated with δ^13^C and positively correlated with leaf total nitrogen concentration, indicating that better growth performance was probably associated with lower WUE and higher nitrogen supply among the tested cultivars. Correlation analysis revealed that several morphological traits, including maximum leaf area, leaf width and length, petiole width and length, leaf number per shoot, and final shoot height, were correlated with shoot fresh biomass. Several physiological traits, namely, leaf δ^13^C, leaf total nitrogen concentration, and water content were also influential factors of biomass production. These results indicate that the variation in shoot biomass is closely associated with several physiological and morphological traits, but the influence of the latter is dominant. Furthermore, LA_max_, leaf δ^13^C, and chlorophyll content (chla and chlb) were identified to be the most important variables for predicting mulberry shoot fresh biomass, accounting for 73% of the variability in shoot fresh biomass. This is the first attempt to link morphological and physiological traits to spring shoot biomass in mulberry. The results indicate that a combination of physiological and morphological traits could be useful in assisting with the early selection of high-yield cultivars in mulberry tree breeding programs. In addition, it is anticipated that the cultivars Xiang7920 and Yunsang2 have higher shoot biomass production in ample or limited water conditions, whereas the cultivars Wupu and Yunguo1, although not superior in biomass production, might have satisfactory biomass production in arid areas.

## 4. Materials and Methods

### 4.1. Plant Material and Growth Conditions

Homogeneous and healthy stem hardwood cuttings (12 cm in length) of eight popular mulberry cultivars—7307, Husang32, Liaolu11, Wupu, Xiang7920, Yu711, Yunguo1, and Yuansang2—were collected from the National Mulberry Germplasm Resource Bank (Zhenjiang, China). The cultivar 7307 is hermaphroditic, the cultivars Liaolu11 and Wupu are male, and the rest of the cultivars are female. The original habitats of these cultivars cover a wide range of agroclimatic conditions in China. The cultivars 7307 and Yu711 originated from Jiangsu Province, and they are suitable for cultivation in Southeast China (Yangtze River area). Xiang7920 originated from Hunan Province and adapts well in Southwest China (Yunnan–Guizhou Plateau) as well as the Yangtze River area. Husang32 was from Zhejiang Province and is widely distributed throughout China. Liaolu11 originated from Liaoning Province in Northeast China. Wupu originated from Shaanxi Province in Northwest China. Yunguo1 and Yunsang2 are generally distributed in Yunnan Province in Southwest China. Adventitious roots were artificially induced on the cuttings by an exogenous application of 200 mg/L indole-3-butyric acid solution as previously described [60]. The well-rooted cuttings were planted with a spacing of 0.5 m × 0.8 m in a botanical garden in the Jiangsu University of Science and Technology (119°23′ E, 32°10′ N; 30 m a.s.l.). The soil was applied with sheep manure (ca. 3.0 kg m^−2^) before transplanting the cuttings, and no more fertilizer was supplied afterward, leaving the trees to grow under natural conditions. This site has a subtropical monsoon climate with an annual precipitation of ca. 1100 mm. The average temperature in spring (from March to May) is 16–20 °C. The ambient CO_2_ concentration is ca. 380 μmol mol^−1^. The saplings were appropriately pruned every year as the applied cultivation routine. The stump was consistently maintained at 60 cm above the ground. All of the discussed investigations were undertaken with 3-year-old trees.

### 4.2. Shoot Morphological and Agronomic Traits Measurements

The mulberry cultivation practice that is routinely applied was used in this study: the mulberry trees were pruned every spring before they began to sprout. Leaf morphological traits were regularly recorded from late April to mid-May as new shoots developed. The length and width of new young leaves (leaf plastochron index, LPI = 3) were recorded in three shoots per tree with a flexible ruler over a period of 3 weeks, and the leaf expansion rate in length (LEX, cm day^−1^) and width (WEX, cm day^−1^) was computed by determining the slope of the linear regression from measurements taken on the first 9 days, when a strong linear relationship was detected. Shoot height, basal diameter, and leaf number per shoot were monitored every three days in three shoots per tree. The shoot height was recorded by a measuring tape, and the shoot diameter was measured with a digital slide caliper from ca. 2 cm above the joint of the main stem and the shoot. One fully illuminated mature leaf that was visually determined to be the largest in the canopy (LPI = 17–21) was collected from each tree and scanned to measure maximal individual leaf area (LA_max_, cm^2^). The leaf area (LA_max_) was estimated by drawing the borders of the scanned leaf image in an image processing tool (Image J, version 1.51w, National Institutes of Health, USA). The length (LL_max_, cm) and width (LW_max_, cm) of the largest leaf were measured with a flexible ruler. Meanwhile, petioles were cut from the leaf, and their length (PL_max_, mm) and width (PL_max_, mm) were measured using a digital slide caliper. Furthermore, specific leaf area of the largest leaf (SLA_max_, cm^2^ g^−1^) was computed as the individual leaf area divided by the leaf dry mass after it was oven-dried at 70 °C for 48 h.

Mulberry trees were subsequently pruned in late May, and the following agronomic traits related to spring shoot biomass were surveyed: total shoot fresh biomass (SFB), leaf fresh biomass of growing shoots (LFB_g_), leaf number per kilogram (LN_pk_), total shoot length (TSL), growing shoots number per tree (SN_pt_), and average shoot length (ASL). Total shoot fresh biomass was measured with an electric scale and calculated as the sum of growing shoots and non-growing shoots. All the leaves on the growing shoots were taken off and weighed for determining foliage production. Mature leaves in the middle parts were weighed to obtain 1 kg of the leaves, which were then counted to determine leaf number per kilogram. The individual length of growing shoots was measured with a measuring tape to record shoot number per tree. Total shoot length was the sum of the individual shoot lengths, and the average shoot length was calculated as the ratio between total shoot length and shoot number per tree.

### 4.3. Leaf Physiological Traits Analysis

Fully expanded and illuminated leaves (LPI=11–13) in each mulberry tree were excised and wrapped in wet paper tissue and immediately carried back to the laboratory. After a brief rinse with tap water, the samples were fast-frozen in liquid nitrogen. Then, the frozen leaves were ground into fine powder in liquid nitrogen with a mortar and pestle before storage at −80 °C. Aliquots of the homogenized samples (ca. 300 mg) were oven-dried at 70 °C for 72 h to determine the foliar water content (WC), which was calculated as follows: WC% = (Fw − Dw)/Fw × 100, where Fw and Dw refer to fresh weight and dry weight of the samples, respectively. The oven-dried samples were further used to assay δ^13^C and δ^15^N as described previously [38]. Briefly, samples were first sifted through a fine mesh to remove any inhomogeneity. Around 1 mg of the aliquoted fine powder was converted into CO_2_ by complete combustion at 850 °C for at least 4 h with excess copper oxide and silver wool in sealed quartz ampoules embedded in an elemental analyzer (Flash EA 2000 HT, Thermo Fisher Scientific Inc., USA). The ratio of ^13^C to ^12^C in the extracted gas was further determined in a coupled gas-isotope-ratio mass spectrometer (Finnigan Delta V Advantage, Thermo Fisher Scientific Inc., USA). Stable carbon isotope composition (‰) was expressed as δ^13^C = (R_sa_ − R_sd_)/R_sd_ × 1000 in δ-unit notation, where R_sa_ is the ratio of ^13^C to ^12^C in the sample and R_sd_ is the ratio in the Pee Dee Belemnite standard (PDB). Protein standards were run regularly during the tests to calibrate any drift error of instrument. Analysis of δ^15^N was similar to that of δ^13^C, with the N_2_ in air used as the standard. The analytic precision was ± 0.1‰ and 0.2‰ for δ^13^C and δ^15^N, respectively. Total carbon and total nitrogen concentrations (%) of each sample were also estimated using the relative peak area of the sample used to determine the abundance of ^13^C and ^15^N compared with a working standard.

Photosynthetic pigment quantification was determined spectrophotometrically according to Wellburn [61]. Briefly, approximately 50 mg of the frozen leaf sample was extracted with 10 mL of 80% acetone. The solution containing the leaf samples was shaken at room temperature in the dark until no green color was visible. The absorbance of the isolated chlorophyll solution was determined at 470, 646, and 663 nm against 80% acetone as a blank with a UV–VIS spectrophotometer (795, APL Instrument Co. Ltd., Shanghai, China). The contents of chlorophyll a, chlorophyll b, total chlorophyll, and carotenoids were computed as previously described [38] and expressed as μg g^−1^ of fresh weight.

### 4.4. Statistical Analysis

To examine the effect of cultivars on experimental variables, all variables were statistically analyzed with one-way ANOVA in Statgraphics (STN, St Louis, MO, USA) and graphed with OriginPro 8 SR0 (version 8.0724, OriginLab Corp., MA, USA). The data distribution was assessed for normality and transformed logarithmically when necessary before performing statistical analysis. Differences in the means between cultivars were considered significant if the *p*-values of the *F*-test were less than 0.05. A correlation test using Pearson’s correlation coefficient was conducted to evaluate the associations among all the morphological and physiological traits. Linear associations between SFB and all the measured traits were also determined. To identify morphological and physiological traits most closely associated with the spring shoot biomass of mulberry trees, a model averaging technique using Akaike’s information criterion (AIC), which calculates the difference in the fit with respect to the complexity of a model, was adopted as previously reported [15]. Among the agronomic traits examined, SFB was used as a response variable to represent spring yield. It is preferred over leaf fresh biomass of growing shoots (LFB_g_) because SFB is the most essential trait in spring. Further, LFB_g_ and SFB were highly correlated (Table 3). Candidate predictive variables were detected from a correlation test and principal component analysis (PCA). In the multiple regression analysis, the only variables selected as predictor variables were those that (1) were not related to each other, (2) were not derived from each other, and (3) had a high contribution to the variations in the investigated traits among the eight cultivars in PCA. Additionally, morphological and physiological traits that exhibited a significant correlation with SFB were included. Briefly, among the 11 morphological and 9 physiological traits investigated in the present study, a union of 12 were used for this analysis for their generally weak correlation with each other and strong contribution to cultivar variations (Figure 5a and Figure S1). For shoot morphological traits, only petiole width (PW_max_), leaf width expansion rate (WEX), maximum leaf area (LA_max_), leaf number per shoot (LN_ps_), shoot height (SH), and shoot diameter (SD) were used. For leaf physiological traits, the following variables were included: stable carbon isotope composition (δ^13^C), foliar water content (WC), and contents of chlorophyll a (chla) and b (chlb). Subsequently, the above-mentioned explanatory variables were standardized and computed by the MuMln package, and the dredge and model.avg functions were used to obtain the best-ranked models. The second-order AIC (AICc) values were used since the sample size was relatively small. The returned best models were specified using a confidence interval at 95%. The correlation test and multiple regression were carried out in R 3.4.4 (R Development Core Team, http://www.rproject.org/).

## Figures and Tables

**Table 1:** Growth-related morphological properties of 3-year-old mulberry trees from eight cultivars. For each variable, the presented data are means (SE). Different letters after the mean values in the same row indicate significant difference at *p* < 0.05. LEX: leaf length expansion rate (cm day^−1^), LEX: leaf width expansion rate (cm day^−1^), LL_max_: maximum leaf length (cm), LW_max_: maximum leaf width (cm), LA_max_: maximum leaf area (cm^2^), PL_max_: maximum petiole length (cm), PW_max_: maximum petiole width (mm), SLA_max_: specific leaf area of the largest leaf (cm^2^ g^−1^), SH: shoot height (m), SD: shoot diameter (mm), LN_ps_: leaf number per shoot.

Variables	Cultivars							
	7307	Husang32	Liaolu11	Wupu	Xiang7920	Yu711	Yunguo1	Yunsang2
LEX	1.32^d^ (0.01)	1.16^c^ (0.04)	1.21^cd^ (0.13)	1.00^b^ (0.07)	1.74^e^ (0.05)	1.31^d^ (0.03)	0.61^a^ (0.07)	1.72^e^ (0.03)
WEX	1.28^cd^ (0.06)	1.37^de^ (0.03)	1.11^c^ (0.13)	0.86^b^ (0.04)	1.58^f^ (0.04)	1.15^c^ (0.01)	0.71^a^ (0.01)	1.46^ef^ (0.02)
LL_max_	18.3^ab^ (1.6)	18.0^ab^ (0.7)	19.3^bc^ (0.8)	19.0^bc^ (0.8)	22.6^cd^ (1.7)	23.4^d^ (1.7)	15.1^a^ (1.4)	24.9^d^ (1.2)
LW_max_	15.8^bc^ (1.0)	14.2^ab^ (0.4)	15.6^b^ (0.7)	14.0^ab^ (0.8)	19.7^d^ (1.8)	18.8^cd^ (1.4)	11.3^a^ (1.0)	21.0^d^ (0.6)
LA_max_	192.2^bc^ (13.4)	165.1^b^ (14.9)	224.4^c^ (21.2)	185.1^bc^ (21.2)	283.8^d^ (20.2)	343.6^e^ (14.5)	104.6^a^ (13.8)	311.1^de^ (30.2)
PL_max_	5.3^c^ (0.2)	4.1^ab^ (0.2)	5.2^c^ (0.3)	5.5^c^ (0.6)	6.7^d^ (0.1)	7.6^d^ (0.6)	3.7^a^ (0.1)	5.1^bc^ (0.1)
PW_max_	3.71^c^ (0.09)	3.40^bc^ (0.16)	3.08^ab^ (0.26)	2.79^a^ (0.08)	4.69^d^ (0.17)	4.53^d^ (0.34)	2.63^b^ (0.19)	4.83^d^ (0.12)
SLA_max_	183.7^c^ (2.0)	178.8^c^ (1.8)	235.5^d^ (6.0)	180.1^c^ (0.1)	152.1^b^ (0.5)	134.5^a^ (1.1)	161.9^b^ (0.8)	157.6^b^ (11.1)
SH	1.04^a^ (0.07)	1.22^bc^ (0.04)	1.60^d^ (0.06)	1.33^c^ (0.05)	1.07^ab^ (0.04)	1.14^ab^ (0.05)	1.35^c^ (0.06)	1.01^a^ (0.07)
SD	16.28^d^ (1.07)	15.67^cd^ (1.04)	20.18^e^ (1.06)	13.54^abc^ (1.05)	14.68^bcd^ (1.09)	14.85^bcd^ (1.05)	12.64^ab^ (1.06)	12.12^a^ (1.05)
LN_ps_	24.2^abc^ (1.3)	21.3^a^ (0.7)	31.5^e^ (1.1)	27.2^cd^ (1.0)	25.5^bc^ (0.7)	22.5^ab^ (0.9)	29.3^de^ (1.1)	26.8^cd^ (1.8)

**Table 2:** Leaf physiological traits: foliar water content (WC, %), chlorophyll a content (chla, mg g^−1^ Fw), chlorophyll b content (chlb, mg g^−1^ Fw), chlorophyll content (chl, mg g^−1^ Fw), carotenoid content (car, μg g^−1^ Fw), stable carbon isotope composition (δ^13^C, ‰), total carbon concentration (C, %), stable nitrogen isotope composition (δ^15^N, ‰) and total nitrogen concentration (N, %) of 3-year-old mulberry trees from eight cultivars. For each variable, the presented data are means (SE). Different letters after the mean values in the same row indicate significant difference at *p* < 0.05.

Variables	Cultivars							
	7307	Husang32	Liaolu11	Wupu	Xiang7920	Yu711	Yunguo1	Yunsang2
δ^13^C	−29.40^b^ (0.05)	−29.59^b^ (0.05)	−30.37^a^ (0.01)	−28.86^cd^ (0.15)	−28.64^d^ (0.06)	−29.07^c^ (0.17)	−28.60^d^ (0.14)	−28.75^d^ (0.05)
C	39.73^a^ (0.3)	43.13^c^ (0.3)	43.95^cd^ (0.03)	43.18^c^ (0.81)	41.53^b^ (0.17)	44.00^d^ (0.83)	44.55^d^ (0.26)	43.95^cd^ (0.15)
δ^15^N	0.88^b^ (0.01)	0.66^b^ (0.01)	2.27^d^ (0.01)	2.45^d^ (0.01)	1.05^bc^ (0.01)	1.75^cd^ (0.01)	0.21^a^ (0.01)	4.40^e^ (0.01)
N	3.11^bc^ (0.02)	2.97^b^ (0.16)	3.87^d^ (0.04)	3.32^c^ (0.21)	2.55^a^ (0.03)	3.03^bc^ (0.01)	3.00^bc^ (0.18)	3.24^bc^ (0.01)
WC	73.7^c^ (0.3)	72.1^bc^ (1.3)	72.2^bc^ (1.3)	68.6^a^ (0.7)	69.7^ab^ (0.9)	68.7^a^ (0.8)	70.6^abc^ (2.0)	67.9^a^ (0.8)
Chla	1.39^cd^ (0.03)	1.19^a^(0.02)	1.49^ed^ (0.02)	1.70^f^ (0.02)	1.28^b^ (0.03)	1.36^c^ (0.01)	1.53^f^ (0.01)	1.44^de^ (0.03)
Chlb	1.07^b^ (0.01)	1.03^ab^ (0.01)	1.16^cd^ (0.05)	1.25^d^ (0.02)	1.00^ab^ (0.01)	1.04^ab^ (0.01)	1.08^bc^ (0.06)	0.97^a^ (0.02)
Chl	2.46^b^ (0.04)	2.22^a^ (0.01)	2.65^c^ (0.06)	2.95^d^ (0.04)	2.28^a^ (0.04)	2.40^b^ (0.01)	2.67^c^ (0.03)	2.41^b^ (0.05)
Car	38.17^b^ (9.08)	4.23^a^ (1.36)	22.27^ab^ (14.5)	39.07^b^ (8.18)	81.67^cd^ (10.13)	33.40^b^ (4.45)	80.20^c^ (4.21)	108.40^d^ (5.67)

**Table 3:** Pearson’s correlation coefficients for the relationship between SFB (shoot fresh biomass) and agronomic, morphological and physiological traits of eight mulberry cultivars. Asterisks indicates the level of significance as *: *p* < 0.05, **: *p* < 0.01 amd ***: *p* < 0.001. LFB_g_: leaf fresh biomass of growing shoots, TSL: total shoot length, SN_pt_: shoot number per tree, ASL: average shoot length, LA_max_: maximum leaf area (cm^2^), LL_max_: maximum leaf length (cm), LW_max_: maximum leaf width (cm), PL_max_: maximum petiole length (cm), PW_max_: maximum petiole width (mm), LN_ps_: leaf number per shoot, SH: shoot height (m), WC: foliar water content (%), δ^13^C: stable carbon isotope composition (‰), N: total nitrogen concentration (%).

Agronomic				Morphological							Physiological		
LFB_g_	TSL	SN_pt_	ASL	LA_max_	LL_max_	LW_max_	PL_max_	PW_max_	LN_ps_	SH	WC	δ^13^C	N
0.89 ***	0.47 *	0.57 **	−0.49 *	0.60 **	0.52 **	0.52 **	0.50 *	0.59 **	−0.45 *	−0.46 *	−0.44 *	0.46 *	−0.43 *

**Table 4:** The regression coefficients for the multiple linear model of shoot fresh biomass (SFB) as a function of maximum leaf area (LA_max_), carbon isotope composition (δ^13^C), and contents of chlorophyll a (chla) and chlorophyll b (chlb). *: *p* < 0.05; **: *p* < 0.01; ***: *p* < 0.001. Residual standard error: 0.7793 with 19 degrees of freedom (DF). Multiple R^2^ = 0.7287, adjusted R^2^ = 0.6716. F-statistic: 12.76 with 4 and 19 DF. The multiple linear regression equation for SFB is y = 46.418 + 0.010LA_max_ + 1.559δ^13^C − 6.404chla + 8.311chlb, *p* = 3.286 × 10^−^^5^.

Variables	Estimate	SE	*t* Value	*p* Value
(Intercept)	46.418	9.337	4.971	8.48 × 10^−5^ ***
LA_max_	0.010	0.002	4.627	0.000184 ***
δ^13^C	1.559	0.327	4.761	0.000136 ***
chla	−6.404	1.728	−3.705	0.001501 **
chlb	8.311	2.958	2.810	0.011185 *

## Supplementary Materials

The following are available online at https://www.mdpi.com/2223-7747/8/5/118/s1, Table S1 Loading of PCA among the eight examined mulberry cultivars. Figure S1 Correlation matrix of selected morphological and physiological traits among the eight examined mulberry cultivars.
