# Supplementary figures and images for: Determinants of Shoot Biomass Production in Mulberry: Combined Selection with Leaf Morphological and Physiological Traits
Source: Plants (Basel). 2019 May 6;8(5):118. doi: 10.3390/plants8050118 (PMC6571901; doi:10.3390/plants8050118)

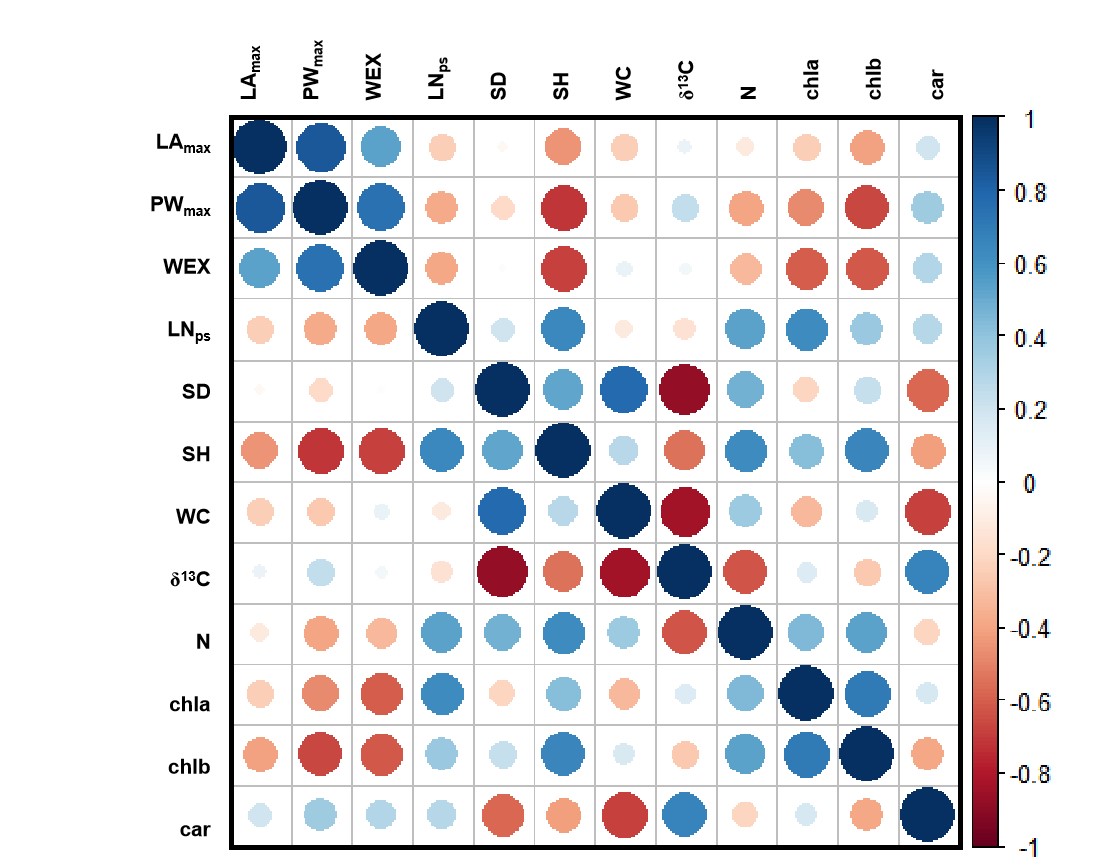

Supplement: Supplementary file 1 [file plants-08-00118-s001.zip › supplementary/plants-471873--.jpg]
